# Spread of Kyasanur Forest Disease, Bandipur Tiger Reserve, India, 2012–2013

**DOI:** 10.3201/eid1909.121884

**Published:** 2013-09

**Authors:** Devendra T. Mourya, Pragya D. Yadav, K. Sandhya, Shivanna Reddy

**Affiliations:** National Institute of Virology, Pune, India (D.T. Mourya, P.D. Yadav);; Virus Diagnostic Laboratory, Shimoga, India (V.K. Sandhya, S. Reddy)

**Keywords:** Kyasanur Forest disease, Shimoga, Karnataka, monkey, outbreak, ticks, viruses, zoonoses, human, India, Bandipur Tiger Reserve

**To the Editor:** Kyasanur Forest disease virus (KFDV; family *Flaviviridae*, genus *Flavivirus*) was first recognized in 1956 in Shimoga District, Karnataka State, India (*1*). The natural cycle of KFDV involves 2 monkey species—black-faced langurs (*Semnopithecus entellus*) and red-faced bonnet monkeys (*Macaca radiata*)—and various tick species (genus *Haemaphysalis*). Monkeys become infected with KFDV through the bite of infected ticks; the virus is then transmitted to other ticks feeding on infected monkeys. KFDV infection causes severe febrile illness in some monkeys. When infected monkeys die, ticks drop from the body, thereby generating hot spots of infectious ticks that further spread the virus. In the enzootic state, KFDV circulates through small mammals (e.g., rodents, shrews, ground birds) and ticks (*2*). 

Humans can also be infected with KFDV. In humans, the disease causes high fever, frontal headache, and severe myalgia, followed by bleeding from the nasal cavity, throat, gingivae, and, in some cases, gastrointestinal tract (*3*). In the natural KFDV cycle, humans are dead-end hosts.

KFD is unique to 5 districts (Shimoga, Chikkamagalore, Uttara Kannada, Dakshina Kannada, and Udupi) in the Malnad region of Karnataka State, India, where each year during January–May, 100–500 persons are affected by the disease (*2*,*4*). During December 2011–March 2012, a total of 215 suspected KFD case-patients were identified in 80 villages in Shimoga District; laboratory testing confirmed that 61 (28%) were infected with KFDV (*5*). 

In November 2012, the deaths of 12 monkeys in Bandipur National Park, Chamarajanagara District, Karnataka State, were reported. At the same time, 6 humans from Mole Hole village and Madhur colony in the Bandipur Tiger Reserve who handled and incinerated the sick monkeys were reported to have clinical signs and symptoms typical of KFD (Technical Appendix Figure 1). The monkey handlers (20–55 years of age) were admitted to the local hospital in Gundlupet Taluk. Monkey autopsy specimens, serum samples from suspected human case-patients, and tick pools were collected by staff from the Virus Diagnostic Laboratory in Shimoga. The samples were sent to the National Institute of Virology in Pune for determination of the etiologic agent. Additional samples from humans with suspected KFDV infection, monkeys, and tick pools were received from Chamarajanagar District and adjoining border areas of Tamil Nadu State and Kerala State (Table).

**Table:** Real-time reverse transcription PCR and nested reverse transcription PCR results for specimens screened for Kyasanur Forest disease virus, India, November 2012–May 2013*

**Date of sample collection**	**Location of sample collection**	**No. samples positive/no. total**		
		Human	Monkey	Tick pools
**2012 Nov**	Maddur Forest Range, Bandipur Tiger Reserve, Chamarajanagara District, Karnataka State	4/6	3/7	–
**2013 Jan**	Chamarajanagara District, Karnataka State	7/13	–	0/7
**2013 Jan**	Nilgiri, Tamil Nadu State	0/1	1/2	0/5
**2013 Feb**	Chamarajanagara District, Karnataka State	–	–	1/2
**2013, May**	Wayanad District, Kerala State	1/1	–	–
**Total no. positive samples**		12/21	4/9	1/14

*–, no samples from the area.

Monkey brain and liver and tick pools were sonicated in 600 μL of Minimum Essential Media (GIBCO/BRL, Life Technologies, Grand Island, NY, USA), and 400 μL of media was added to the homogenate. TriPure Isolation Reagent (Roche Diagnostics, Indianapolis, IN, USA) was used to perform RNA extraction as described (*6*).

Samples were tested for KFDV by nested reverse transcription PCR (RT-PCR) and real-time RT-PCR as described (*6*); 12 of 21 human samples and 4 monkey samples were positive (Table). Two of 14 tick pools screened for KFDV by real-time RT-PCR were positive; however, 1 was weakly positive (Table). The PCR-amplified products were purified by using the QIAquick Gel Extraction Kit (QIAGEN, Hilden, Germany) and then sequenced. KFDV sequences from the samples showed 95.8%−98.1% similarity with prototype strain KFDV P9605. This finding supports the earlier conclusion that a high level of conservation exists for KFDV sequences (*7*). The phylogenetic tree formed 2 clades: the first included mainly KFDV sequences from 1957–2006, the second included KFDV sequences (human and monkey) from Chamarajanagara District (Technical Appendix Figure 2).

KFDV has not been detected previously in Chamarajanagara District, the location of Bandipur National Park. Affected areas in the district share a border with Mysore District (Karnataka State), Kerala State, and Tamil Nadu State. In addition,we subsequently found monkey samples from Nilgiri, Tamil Nadu, to be positive for KFDV.

The human case-patients from Chamarajanagara District were mainly forest workers involved in the incineration of the dead monkeys. Infection among these workers indicates that they did not follow appropriate biosafety procedures while handling the infected animals.

Our findings confirm that KFD has occurred outside the districts in Karnataka State where KFDV is known to be endemic. A hemagglutination inhibition antibody survey conducted during December 1988–January 1989 (*8*) indicated the possible existence of this disease in other regions of India. The presence of KFD becomes noticeable when enzootic infections occur and sentinel animals, like monkeys, start dying (*9*). Detection of KFDV in Chamarajanagara District, Tamil Nadu State (Nilgiri), and Kerala State indicates the presence of the virus in many evergreen and semi-evergreen forest areas of India. Infections in these areas may have been missed previously because of the lack of an organized surveillance system.

During the first week of December 2012, immediately after the KFD outbreak was confirmed, the Karnataka public health department vaccinated 322 persons, including villagers, forest officials, health workers, and members of local tribes in the Maddur Forest Range of Bandipur Tiger Reserve. Hot-spot areas caused by monkey deaths were dusted with malathion insecticide to kill ticks. In addition, to prevent additional human infections, epidemiologists recommended establishment of a health education campaign and the use of protective clothing and tick repellents, especially by persons frequently visiting forested areas.

Technical Appendix Real-time reverse transcription PCR and reverse transcription PCR results for specimens tested for Kyasanur Forest disease virus (KFDV), new KFD outbreak areas in Karnataka State, India, during 2012–2013, and phylogenetic analysis of KFDV sequences.
