# Supplementary material for: Spread of Kyasanur Forest Disease, Bandipur Tiger Reserve, India, 2012–2013
Source: Emerg Infect Dis. 2013 Sep;19(9):1540–1. doi: 10.3201/eid1909.121884 (PMC3810911; doi:10.3201/eid1909.121884)
Supplement: Technical Appendix — Real-time reverse transcription PCR and reverse transcription PCR results for specimens tested for Kyasanur Forest disease virus (KFDV), new KFD outbreak areas in Karnataka State, India, during 2012–2013, and phylogenetic analysis of KFDV sequences. [file 12-1884-Techapp-s1.pdf]

Article DOI: http://dx.doi.org/10.3201/eid1909.121884

Spread of Kyasanur Forest Disease, Bandipur Tiger Reserve, India, 2012–2013

Technical Appendix

Technical Appendix Table. Results of screening for Kyasanur Forest disease virus in human specimens, monkey specimens, and tick pools from Karnataka, Kerala, and Tamil Nadu States, India, December 2012–May 2013*

Specimen type, ID no.	Date sample collected	Specimen type	Age of human or monkey	Location‡	rRT-PCR result, C_t	RT-PCR result	Final result
Human†							
1221197	Nov2012	Serum	22 y	Madduru Forest Range, Chamarajanagara	38.5	+	+
1221199	Nov 2012	Serum	44 y	Madduru Forest Range, Chamarajanagara	38.7	–	+
1221200	Nov 2012	Serum	46 y	Madduru Forest Range, Chamarajanagara	34.7	+	+
1221201	Nov 2012	Serum	55 y	Madduru Forest Range, Chamarajanagara	27.5	+	+
13192	Jan 2013	Serum	28 y	Chamarajanagara	33.5	+	+
131149	Jan 2013	Blood	60 y	Chamarajanagara	33.5	–	+
131150	Jan 2013	Blood	35 y	Chamarajanagara	30.5	–	+
131151	Jan 2013	Blood	45 y	Chamarajanagara	26.5	+	+
131152	Jan2013	Blood	60 y	Chamarajanagara	28.0	+	+
131153	Jan 2013	Blood	43 y	Chamarajanagara	28.5	+	+
131154	Jan 2013	Blood	19 y	Chamarajanagara	33.5	–	+
135724	May 2013	Blood	18 y	Wayanad District, Kerala State	30.0	+	+
Monkey†							
AN1221204-1	Nov 2012	Brain, liver	Adult	Halegoudana camp, Chamarajanagara	36.5<br>38.0	–	+
AN1221207-3	Nov 2012	Brain, liver	Adult	Halegoudana camp, Chamarajanagara	22.20<br>22.80	+	+
AN1221208	Nov 2012	Brain	Adult	Halegoudana camp, Chamarajanagara	36.40	+	+
AN131058	Nov 2012	Brain	Adult	Nilgiri, Tamil Nadu State	26.80	+	+
Tick 114	Feb 2013	Hyalomma mixed tick pool		Chamarajanagara	22.00	+	+

*Screening was performed by the National Institute of Virology in Pune, India. rRT, real-time reverse transcription; ID, identification; C_t, threshold cycle.
†All humans and monkeys were male.
‡Chamarajanagara is a district in Karnataka State.

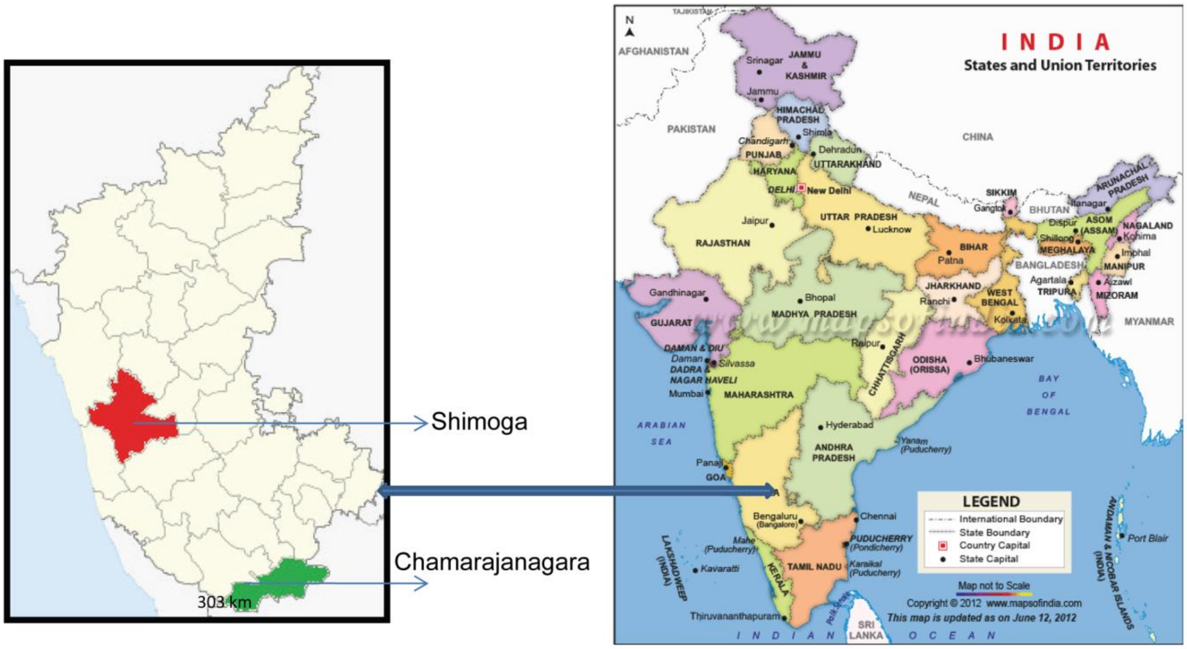

Technical Appendix Figure 1. Kyasanur Forest disease outbreak in new area of Karnataka State, India, 2012–2013. Map source: www.mapsofindia.com.

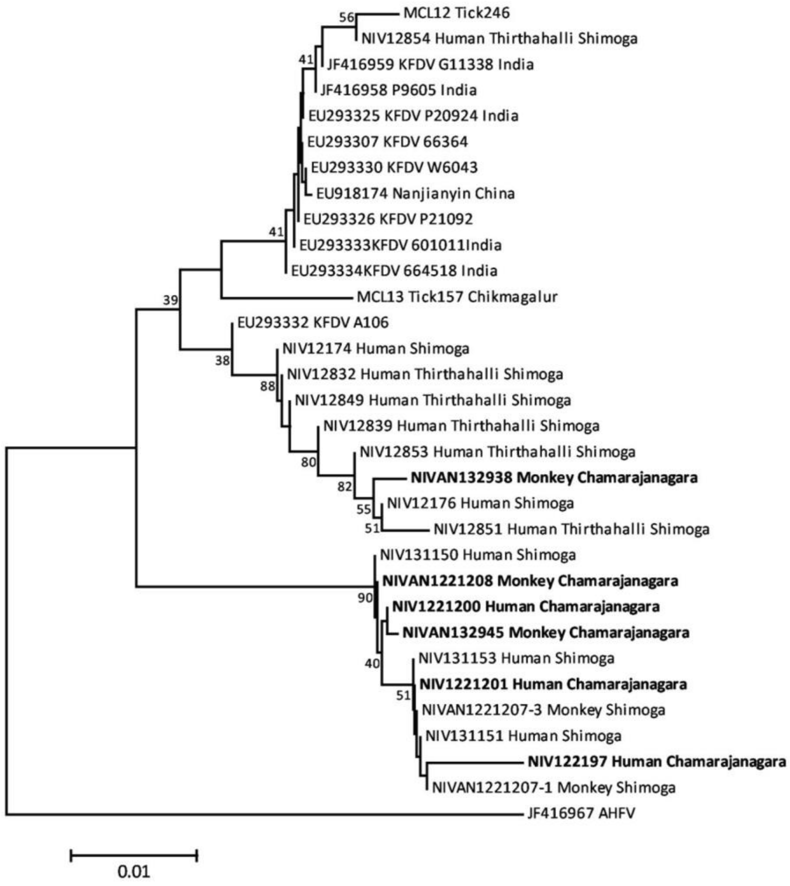

Technical Appendix Figure 2. Phylogenetic analysis of Kyasanur Forest disease virus sequences from recent outbreak and earlier Kyasanur Forest disease virus sequences from Karnataka State, India.
